# Estimating a change-point of baseline age in the longitudinal trajectories of biomarkers: application to an imaging study of preclinical Alzheimer disease

**DOI:** 10.21203/rs.3.rs-6681661/v1

**Published:** 2025-06-12

**Authors:** Chengjie Xiong, Folasade Agboola, Jingqin Luo

**Affiliations:** 1. Division of Biostatistics, Washington University School of Medicine, St. Louis, MO, USA; 2. Knight Alzheimer Disease Research Center, Washington University School of Medicine, St. Louis, MO, USA.; 3. Division of Public Health Sciences, Department of Surgery, Washington University School of Medicine, St. Louis, MO, USA; 4. Siteman Cancer Center Biostatistics and Qualitative Research Shared Resource, Washington University School of Medicine, St. Louis, MO, USA

**Keywords:** Alzheimer disease, biomarker, change-point, point and confidence interval estimators

## Abstract

**Background:**

Biomarkers are routinely measured from human biospecimens and imaging scans in Alzheimer disease (AD) research. Age is a well-known risk factor for AD. Detecting the age at which the longitudinal change in biomarkers starts to accelerate, i.e., a change-point in age, is important to design preventive interventions.

**Methods:**

We analyzed longitudinal biomarker data by a random intercept and random slope model where the slope (longitudinal rate of change) was modeled as a piecewise linear and continuous function of baseline age. We proposed to estimate the intersection of the two linear functions, i.e., the change-point in age by multiple methods: maximum (profile) likelihood, minimum squared pseudo bias, minimum variance, minimum mean square error (MSE), and a two-stage method. We simulated large numbers of data sets to evaluate the performance of these estimators and implemented them to analyze the longitudinal white matter hypointensity from brain magnetic resonance imaging scans in an AD cohort study of 616 participants to estimate the age when the longitudinal rate of change starts to accelerate.

**Results:**

Our simulations indicated that performance was universally poor for all point estimators and CI estimates when the true change-point was near the boundary or when sample size was small (N=100). Yet, the proposed change-point estimators became approximately unbiased and showed relatively small MSE when sample size increased (N>200) and the true change-point was away from boundary. The 95% CIs from these methods also provided good nominal coverage with large sample sizes if the change-point was away from boundary. When applied to the AD biomarker study, we found that almost all methods yielded similar estimates to the change-point from 59.19 years to 65.78 years, but the profile likelihood approach led to a much later estimate.

**Conclusions:**

Our proposed estimators for the change-point performed reasonably well, especially when it is away from the boundary and the sample sizes are large. Our methods revealed a largely consistent age when the longitudinal change in white matter hypointensity started to accelerate. Further research is needed to tackle more complex challenges, i.e., multiple change-points that may depend on other AD risk factors.

## Background

**Figure 1.**
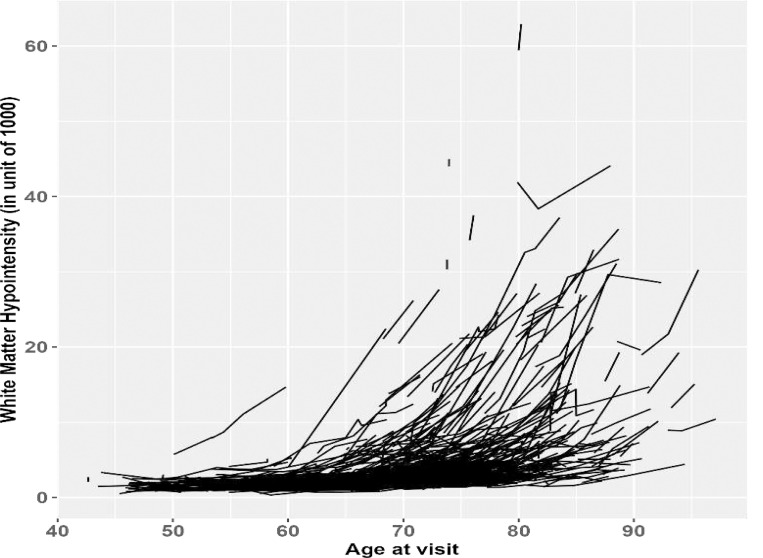
Longitudinal spaghetti plot of brain MRI white matter hypointensities (in unit of 1000) as a function of age on N=616 cognitively normal participants from Washington University Knight AD Research Center. Each spaghetti represents longitudinal data from an independent participant. Participants with younger baseline age exhibit relatively flat trajectories in contrast to older individuals with an accelerated rate of increase.

Longitudinal biomarkers, including those measured from human biospecimens and imaging scans (e.g., brain magnetic resonance imaging [MRI]) scans), are routinely collected to track the evolution of disease processes. Age is a major risk factor for many neurodegenerative diseases, for example, Alzheimer disease (AD). Because the neurodegenerative processes of AD may begin years or even decades prior to the onset of clinical and cognitive symptoms^1^, prevention trials of AD must determine the baseline age interval to enroll asymptomatic individuals who are at elevated risk of AD. One way to identify such high-risk individuals is to pinpoint the age when the longitudinal rates of change in AD biomarkers start to accelerate. For example, white matter hyperintensities (WM-hyper) and hypointensities (WM-hypo) measure signal abnormalities in the brain that can be visualized in the cerebral white matter on different types of MRI scans and sequences. WM-hyper and WM-hypo are highly correlated with each other^2^, and both are associated with validated and established biomarkers of AD and cognitive decline^3^, especially among individuals who are cognitively normal. Figure 1 presents the spaghetti plot of 616 cognitively normal individuals at baseline from Washington University (WU) Knight Alzheimer Disease Research Center (ADRC) whose WM-hypo volumes were longitudinally measured. Because ~79% of the 616 participants had only two to four longitudinal assessments, a linear growth model is reasonable to capture the within-individual longitudinal change, which implies that the random intercept and random slope model^4^ may fit the longitudinal data well. Figure 1 clearly indicates that the longitudinal rate of change in WM-hypo, i.e., the slope in the model, depends on baseline age in the sense that younger individuals at baseline showed minimal increases over time, whereas older individuals demonstrated an accelerated rate of increase over time. Of significant clinical interest is the location of the age at baseline when the longitudinal rate of change in WM-hypo starts to accelerate because this age may determine the inclusion criterion of participant enrollment for future prevention trials on AD.

The statistical challenge here is to accurately estimate the age at baseline when the longitudinal profile of the biomarker starts to diverge, and this age is often called a change-point, or an inflection point or a break-point. The estimation of a change-point has been studied by many authors. The initial work was in the time series field where a sudden shift in mean or variance is the main interest. Change-point detection in a cross-sectional setting with general linear regression models has also been studied ^5,6^ with a primary goal of identifying break-points at which the functional form of the estimated regression models starts to change. Longitudinal data are crucial in understanding the natural history of the trajectories of diseases such as AD, and detecting one or more change-points in the middle of longitudinal follow up has also been studied in HIV infection and cognitive decline^7–11^, both in univariate and multivariate settings^9^‘^12^. However, these models only apply to studies of very long duration in longitudinal follow up with many frequent within-individual repeated assessments over time because longitudinal data prior to and after the change-point must be available to estimate a change-point in the longitudinal and nonlinear trajectories of biomarkers. Whereas Figure 1 does represent longitudinal data in WM-hypo across a very wide age span across subjects at baseline from mid-40s to almost 100 years, the within-subject longitudinal follow up is short and the frequency of repeated measurement is low and mostly from 2 to 4 time points.

**Figure 2.**
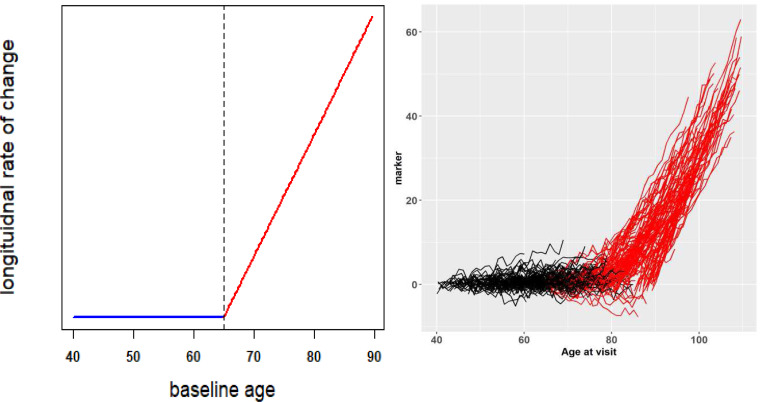
A schema of the proposed model for the longitudinal rate of change and simulated longitudinal trajectories. In the left panel, the longitudinal slope is modeled as a continuous piecewise linear function of baseline age. In the right panel, subject-level longitudinal trajectories simulated from the model in the left panel are visualized as functions of age. The colors of black and red differentiate subjects with baseline age prior to and post the change-point.

In this paper we leverage the longitudinal design feature as represented in Figure 1 and address the statistical challenge of detecting a change-point in age at baseline that the longitudinal rate of change in WM-hypo starts to accelerate. Because of the short follow up and low frequency of within-subject repeated measurements, we conceptualize a random intercept and random slope model for the longitudinally assessed AD biomarker. Because of the very long age span at baseline across the subjects, we further conceptualize that the longitudinal rate of change is a nonlinear function of baseline age, which is approximated by a piecewise linear function (Figure 2, left panel). The conceptualization is directly motivated by Figure 1 which demonstrates that, after some baseline age, e.g., the change-point, the longitudinal slopes of the AD biomarker start to accelerate, in comparison to the longitudinal slope of the individuals whose baseline age is younger than the change-point.

The rest of this paper is organized as follows. We give details of our conceptualized longitudinal model in Section 2. We then propose various methods to estimate the change-point and construct the confidence intervals. Simulation studies are conducted to compare the proposed estimators in Section 3. In Section 4, we return to the real-world AD example as presented in Figure 1 and apply the proposed algorithms to estimate the change-point in baseline age for WM-hypo. We conclude the paper with discussions in Section 5.

## Methods

### A longitudinal model

Let $y_{ij}=y\left(t_{ij}\right)$ denote the longitudinal measurement of a biomarker (e.g., WM-hypo in Figure 1) on subject i, (i=1,2,..,N) at time $t_{ij}$ with $t_{ij}=0$ at baseline. Because many of the AD biomarkers are subject to relatively short and limited number of follow-ups, a within-subject linear growth trend over time is often reasonable, as demonstrated in Figure 1. The longitudinal measurements $y_{ij}$ can then be modeled by a random intercept and random slope model to incorporate the linear trend parameterized by the fixed intercept and fixed slope, as well as subject-level deviations from the overall trend by subject-level random effects on intercepts and slopes. We further model the fixed slope as a function of baseline age with a change-point $\left(\tau \right)$, such that, as illustrated in Figure 2 (left panel), the longitudinal rate of change (i.e., the slope) constitutes two piecewise linear functions of baseline age, depending on whether the baseline age of a subject is older or younger than τ.

Specifically, the proposed longitudinal change-point model can be written as (for the simplicity of notations, no other covariates than baseline age is included in the fixed effects, but the model can readily allow other covariates in both fixed intercept and slope),

(Model 1) $$y_{ij}=\left\{\begin{array}{ll} \beta_{0-}+b_{0-i}+\left[\beta_{1}+\beta_{1-}\times \left(age_{i}-\tau \right)+b_{1-i}\right]\times {\text{t}}_{ij}+\varepsilon_{ij-} & \text{if}\;age_{i}<\tau \\ \beta_{0+}+b_{0+i}+\left[\beta_{1}+\beta_{1+}\times \left(age_{i}-\tau \right)+b_{1+i}\right]\times {\text{t}}_{ij}+\varepsilon_{ij+} & \text{if}\;age_{i}\geq \tau \end{array}\right.$$

where $age_{i}$ is the baseline age of subject i and $t_{ij}$ is the time since baseline (=0 at baseline), $\beta_{1}$ is the longitudinal slope for the subjects of baseline age at the change-point τ, $\beta_{0-}$ and $\beta_{0+}$ are the intercepts for subjects whose baseline age is prior to and post the change-point, respectively. $\beta_{1-}$ and $\beta_{1+}$ represent the increment in the longitudinal slope (as linear functions of baseline age,$age_{i}$) corresponding to each one year’s (at baseline) contribution to the longitudinal slope, for subjects whose baseline age is younger or older than the change-point, respectively. Whereas the intercepts, $\beta_{0-}$ and $\beta_{0+}$, can also be modeled as continuous functions of baseline age with $\beta_{0-}(\tau )=\beta_{0+}(\tau )$, our primary focus in this paper will be on the slope part of the model as a function of baseline age. Thereafter, we will use $\beta_{0-}$ and $\beta_{0+}$ to denote the set of parameters from the intercept parts of the model. Let $\beta =\left(\beta_{0-},\beta_{0+},\beta_{1},\beta_{1-},\beta_{1+}\right)$ be the fixed-effect regression parameters. The subject-level random intercept and random slope pairs $\left(b_{0-i},b_{1-i}\right)$ and $\left(b_{0+i},\right.\left.b_{1+i}\right)$ are independent of each other and each follows a bivariate normal distribution, $\left(b_{0-i},b_{1-i}\right)∼N\left(0,{\text{Σ}}_{-}\right)$ and $\left(b_{0+i},b_{1+i}\right)∼N\left(0,{\text{Σ}}_{+}\right)$, with ${\text{Σ}}_{-}$ and ${\text{Σ}}_{+}$ being distinct unstructured covariance matrices, reflecting that the variance/covariance of the random effects may differ prior to and post the change-point, as observed from Figure 1. ${\varepsilon_{ij-}}^{\prime }s$ are independent and normally distributed as $N\left(0,\sigma_{-}^{2}\right)$, and ${\varepsilon_{ij+}}^{\prime }s$ are independent and normally distributed as $N\left(0,\sigma_{+}^{2}\right)$. Further, we make the other standard assumptions that the random effects $\left(b_{0-i},b_{1-i}\right)$ and $\left(b_{0+i},b_{1+i}\right)$ are all independent of $\varepsilon_{ij-}$ and $\varepsilon_{ij+}$, and point out that our estimators proposed in Section 3 can be readily extended to include other covariance structures for the error terms, $\varepsilon_{ij-}$ and $\varepsilon_{ij+}$.

To confirm that the proposed model reasonably captures the longitudinal data of MRI WM-hypo in Figure 1 that directly motivated this work, we simulated longitudinal trajectories of individual subjects as visualized in Figure 2 (right panel) where the baseline age was assumed to be uniformly distributed from 40 to 90 years, a baseline age change-point was set at 65 years old, all the intercepts were set at 0, a mean longitudinal slope of 0 prior to the change-point and a mean slope of 0.1 post the change-point (i.e., $\beta_{1}=0.1$, $\beta_{1-}=0$ and $\beta_{1+}=0.1$, using the notations in Model 1). As shown in Figure 2 (right panel), the simulated longitudinal trajectories reasonably captured the longitudinal pattern observed in the spaghetti plot of the MRI WM-hypo (Figure 1) in the real-world data set.

### Maximum Likelihood Estimate (MLE) via Profile Likelihood

Both the change-point τ, and the other parameters in Model (1), collectively denoted by $\text{φ}=\left(\text{β},\Sigma_{-},\Sigma_{+},\sigma_{-}^{2},\sigma_{+}^{2}\right)$, can be estimated by maximizing the joint log likelihood $l(\tau ,\varphi )=\sum_{i}\sum_{j}log\;f\left(y_{ij};\tau ,\varphi \right)$ of Model (1): $\left(\hat{\tau },\hat{\varphi })=\arg\;max_{\tau ,\varphi }\;l(\tau ,\varphi \right)$. Because τ is the parameter of central interest here, we can estimate τ by profiling out φ using the profile likelihood method. Assume that τ is known, the joint log likelihood l(τ,φ) is then denoted by $l_{\tau }(\varphi )$, and we can estimate ${\hat{\varphi }}_{\tau }=\arg\;max_{\varphi }\;l_{\tau }(\varphi )$. Plugging in ${\hat{\varphi }}_{\tau }$ into the joint log likelihood gives the profile likelihood $l_{\tau }(\hat{\varphi })=l\left(\tau ,{\hat{\varphi }}_{\tau }\right)$. We can ultimately obtain $\hat{\tau }$ as the one maximizing all the $l_{\tau }(\hat{\varphi })$, i.e., ${\hat{\tau }}_{maxLogLike}=\arg\;max_{\tau }\;l\left({\hat{\varphi }}_{\tau }\right)$ via a grid search among all possible change-points. The resultant profile likelihood maximizing estimators $\left({\hat{\tau }}_{maxLogLike},{\hat{\varphi }}_{\hat{\tau }}\right)$ are known to also maximize the joint log likelihood l(τ,φ). The joint likelihood function at the change-point is non-differentiable at τ and thus the asymptomatic variance cannot be directly derived. However, the 95% profile confidence interval (labeled as $CI_{profileCI}$) to τ can be readily obtained^13^.

### Another Point Estimator of the Change-point

In addition to the profile MLE, we propose several alternative approaches to estimate the change-point. Given the true change-point τ, the subjects with baseline age prior to τ will be assigned to an index set, ${\text{Ω}}_{-}=\left\{i|age_{i}<\tau ,i=1,2,\ldots ,N\right\}$ and those with baseline age post τ belong to the index set ${\text{Ω}}_{+}=\left\{i|age_{i}\geq \tau ,i=1,2,\ldots ,N\right\}$. We can separately fit a random intercept and random slope model to subset ${\text{Ω}}_{-}$ as,

(Model 2.1) $$y_{ij}=\left(\alpha_{0-}+a_{0i-}\right)+\left(\gamma_{0-}+\gamma_{1-}age_{i}+b_{1i-}\right)\times {\text{t}}_{ij}+\varepsilon_{ij-}$$

and to subset ${\text{Ω}}_{+}$ as,

(Model 2.2) $$y_{ij}=\left(\alpha_{0+}+a_{0i+}\right)+\left(\gamma_{0+}+\gamma_{1+}age_{i}+b_{1i+}\right)\times {\text{t}}_{ij}+\varepsilon_{ij+}.$$

Note that Model (1) is nested within Models (2.1) and (2.2) in that the latter do not require that the longitudinal slope, as a function of baseline age, be continuous at the change-point, which is the reason we used a different set of fixed effect parameters in Model (2.1) and Model (2.2). Here $\left(\gamma_{0-}+\gamma_{1-}age_{i}+b_{1i-}\right)$ and $\left(\gamma_{0+}+\gamma_{1+}age_{i}+b_{1i+}\right)$ are the longitudinal rates of change for the two subsets. $b_{1i-}$ and $b_{1i+}$ are the random effects for the longitudinal slope. $\alpha_{0-}$ and $\alpha_{0+}$ denote the fixed intercepts and $a_{0i-}$ and $a_{0i+}$ denote the random intercepts for two subsets. The random intercept and slope vectors $\left(a_{0i-},b_{1i-}\right)$ in Model (2.1) and $\left(a_{0i+},b_{1i+}\right)$ in Model (2.2) each independently follow a bivariate normal distribution with a mean vector (0,0) and a *2×2* unstructured variance/covariance matrix, and the error terms $\varepsilon_{ij-}\text{’s}$ and $\varepsilon_{ij+}\text{’s}$ each independently follow a normal distribution with mean 0 and its own variance.

Model (1) is mathematically equivalent to Model (2.1) and (2.2) if an additional constraint is imposed on the continuity for the slope as a function of baseline age in Model (2.1) and (2.2), i.e.,

(3) $$\gamma_{0+}+\gamma_{1+}\times \tau =\gamma_{0-}+\gamma_{1-}\times \tau .$$

When $\gamma_{1+}-\gamma_{1-}\neq 0$, we have,

(4) $$\tau =\frac{\gamma_{0-}-\gamma_{0+}}{\gamma_{1+}-\gamma_{1-}}.$$

Hence, if Model (1) is the true model, one can estimate τ by Equation (4), labeled as $\hat{\tau }$, by substituting the parameters by their corresponding MLEs after fitting Model (2.1) and (2.2) independently on two subsets of participants,

(5) $$\hat{\tau }=\frac{{\hat{\gamma }}_{0-}-{\hat{\gamma }}_{0+}}{{\hat{\gamma }}_{1+}-{\hat{\gamma }}_{1-}}.$$

However, $\hat{\tau }$ is not computable because the true change-point τ and the two index sets are unknown and the parameters $\gamma =\left(\gamma_{0-},\gamma_{1-},\gamma_{0+},\gamma_{1+}\right)$ cannot be estimated from Model (2.1) and Model (2.2) without knowing τ. We propose to obtain estimates to τ by a grid search algorithm as detailed later in Section 3.4. In the subsequent Section 3.3, we again assume τ is given, and discuss the variance and the sampling distribution of $\hat{\tau }$, which then lead to confidence interval (CI) estimates to τ. Note that if τ is given, the MLE estimates of the parameters in γ, $\hat{\gamma }$, follows an asymptotic normal distribution with mean γ and variance/covariance matrix given by

$$Var(\hat{\gamma })=\left(\begin{array}{ccc} Var\left({\hat{\gamma }}_{0-}\right) & Cov\left({\hat{\gamma }}_{0-},{\hat{\gamma }}_{1-}\right) & 0\;0\\ Cov\left({\hat{\gamma }}_{0-},{\hat{\gamma }}_{1-}\right) & Var\left({\hat{\gamma }}_{1-}\right) & 0\;0\\ 0 & 0 & Var\left({\hat{\gamma }}_{0+}\right)\;Cov\left({\hat{\gamma }}_{0+},{\hat{\gamma }}_{1+}\right)\\ 0 & 0 & Cov\left({\hat{\gamma }}_{0+},{\hat{\gamma }}_{1+}\right)\;Var\left({\hat{\gamma }}_{1+}\right) \end{array}\right),$$

which can be estimated after independently fitting Model (2.1) and (2.2).

### Confidence Interval of the Change-point

Assuming τ is given in Model (2.1) and (2.2), we can derive the variance to $\hat{\tau }$, the change-point estimate in Equation (5), using the Delta method as,

$$Var_{delta}(\hat{\tau })=D\times Var(\hat{\gamma })\times D^{\prime }$$

where $D=\left(\begin{array}{c} \frac{1}{{\hat{\gamma }}_{1+}-{\hat{\gamma }}_{1-}},\;\frac{{\hat{\gamma }}_{0-}-{\hat{\gamma }}_{0+}}{{\left({\hat{\gamma }}_{1+}-{\hat{\gamma }}_{1-}\right)}^{2}},\;\frac{-1}{{\hat{\gamma }}_{1+}-{\hat{\gamma }}_{1-}},\;\frac{-\left({\hat{\gamma }}_{0-}-{\hat{\gamma }}_{0+}\right)}{{\left({\hat{\gamma }}_{1+}-{\hat{\gamma }}_{1-}\right)}^{2}} \end{array}\right)$ is the first derivative vector of τ with respect to γ in Equation (4), prime denotes matrix transpose, and all the multiplications are matrix multiplications. Specifically,

$$Var_{delta}(\hat{\tau })=\frac{{\hat{\tau }}^{2}\left[\hat{Var}\left({\hat{\gamma }}_{1+}\right)+\hat{Var}\left({\hat{\gamma }}_{1-}\right)\right]+2\hat{\tau }\left[\hat{Cov}\left({\hat{\gamma }}_{0-},{\hat{\gamma }}_{1-}\right)+Cov\left({\hat{\gamma }}_{0+},{\hat{\gamma }}_{1+}\right)\right]+\left[\hat{Var}\left({\hat{\gamma }}_{0-}\right)+\hat{Var}\left({\hat{\gamma }}_{0+}\right)\right]}{{\left({\hat{\gamma }}_{1+}-{\hat{\gamma }}_{1-}\right)}^{2}}.$$

Hence, $\hat{\tau }$ may be approximated (at least asymptotically) by a normal distribution with mean τ and variance $Var_{delta}(\hat{\tau })$. The 95% Wald CI (labeled as $CI_{delta}(\hat{\tau })$) is

(6) $$\left(\hat{\tau }-1.96\times \sqrt{Var_{delta}(\hat{\tau })},\hat{\tau }+1.96\times \sqrt{Var_{delta}(\hat{\tau })}\right).$$

Note that this Wald CI is consistent with the CI derived previously by Hinkley^14^.

The existence of a change-point can be statistically confirmed by testing the null hypothesis $H_{0}:\gamma_{1+}=\gamma_{1-}$ against the alternative $H_{1}:\gamma_{1+}\neq \gamma_{1-}$, based on a standard Wald test using the estimates to the slope difference and the associated variance after fitting Model (2.1) and (2.2).

### Estimation by a Grid Search

Note that neither the point estimator $\hat{\tau }$ in Equation (5) nor the CIs proposed in Section 3.3 can be computed unless the true change-point is first estimated. We now propose several ways to estimate the change-point. Given a possible and computationally feasible change-point value c which can be taken from a fine grid of all possible values, e.g., the range of baseline age $\left(age_{i}\right)$, all the subjects can be separated into two subsets: one contains all the subjects whose baseline ages are younger than c, and the other contains all the subjects whose baseline ages are at c or older. Model (2.1) and (2.2) can be fitted to the two subsets of subjects, respectively. Using the MLEs from these models, the change-point can be estimated according to Equation (5). The algorithm is detailed below.

1. Define ∪ as the set of possible change-points based on the practical range of baseline age, for example, a fine grid from the 5% quantile to the 95% quantile of baseline age with a granular increment of 0.1. If the 5% and 95% quantile of baseline age is 50 and 70 years, then the set of all possible change-points would be ∪=(50.1,50.2,50.3,50.4,50.5,…,69.7,69.8,69.9,70);
2. For each possible change-point c∈∪, fit Model (2.1) to all the subjects with a baseline age less than c and Model (2.2) to those with a baseline age at c and older to obtain the MLEs to the model parameters;
3. Obtain the change-point estimator at given c by Equation (5) and denote it by ${\hat{\tau }}_{c}$. Compute the squared “pseudo bias” as $BIAS_{pseudo}^{2}\left({\hat{\tau }}_{c}\right)={\left({\hat{\tau }}_{c}-c\right)}^{2}$ and the mean squared error (MSE) of ${\hat{\tau }}_{c}$ as $MSE_{delta}\left({\hat{\tau }}_{c}\right)=Var_{delta}\left({\hat{\tau }}_{c}\right)+BIAS_{pseudo}^{2}\left({\hat{\tau }}_{c}\right)$. Obtain the Delta method-based variance as given in Section 3.3 (denote it by $Var_{delta}\left({\hat{\tau }}_{c}\right)$) and the corresponding CI in Equation (6) (denote it by $CI_{delta}$);
4. Repeat the above steps for all c∈∪.

We now propose a set of point estimators to τ using the following optimization criteria:

1. minimizing the squared pseudo bias (*minBias*): ${\hat{\tau }}_{minBias}=\underset{c\in \cup }{\text{argmin}}\;BIAS_{pseudo}^{2}\left({\hat{\tau }}_{c}\right)$,
2. minimizing the Delta method-based variance (*minVar*) as in Section 3.3 or equivalently minimizing CI length of $CI_{delta}:{\hat{\tau }}_{minVar}=\underset{c\in \cup }{\text{argmin}}\;Var_{delta}\left({\hat{\tau }}_{c}\right)$,
3. minimizing the MSE (*minMSE)*: ${\hat{\tau }}_{minMSE}=\underset{c\in \cup }{\text{argmin}}\;MSE_{delta}\left({\hat{\tau }}_{c}\right)$.

Note that each of the three point estimators proposed here to the change-point τ can be plugged into the Delta-based variance and CI in Equation (6) to obtain a corresponding CI to change-point.

### A Two-Stage Estimator

We finally propose another intuitive estimator to the change-point that can be obtained through a two-stage procedure, similar to the approach of a change-point estimator in a cross-sectional segmented regression setting^5^. At the first stage, a simple random intercept random slope model as below will be fitted to the entire longitudinal data across all subjects,

$$y_{ij}=\alpha_{0}+b_{0i}+\left(\gamma_{1}+b_{1i}\right)\times {\text{t}}_{ij}+\varepsilon_{ij},$$

with the fixed effect intercept $\alpha_{0}$ and slope $\gamma_{1}$ and subject-specific random intercept $b_{0i}$ and random slope $b_{1i}$. At the 2^nd^ stage, the subject-level random slopes $b_{1i}$ estimated from the 1^st^ stage will be regressed on subjects’ baseline age to estimate the change-point using the segmented regression method^5^. Briefly, Muggeo^5^ (see Equation 2 therein) estimated the cross-sectional change-point via an iterative procedure based on the first order Taylor expansion around an initial starting value of the change-point in approximation of the linear regression term. We label the two-stage change-point estimate as ${\hat{\tau }}_{2stage}$. The 95% normal distribution-based CI to the change-point estimator can be constructed using the variance derived in Muggeo^5^ (see Equation 5 therein). Davies test^15,16^ as used in Muggeo ^5^ can be used to test whether there exists a change-point.

## Results

### Simulation Settings

We evaluated the performance of the proposed estimators and variances in Section 3 through an extensive simulation study. The point estimators under evaluation are described in the sections above: *maxLogLike=* maximizing log profile likelihood of Model (1), from the stratified Model (2.1) and (2.2) based on Equation(5): *minBias*=minimizing the squared pseudo bias, *minVar*= minimizing the Delta method-based variance, and *minMSE*=minimizing the MSE (sum of squared pseudo bias and Delta method-based variance), and the 2-stage approach in Section 3.5: *2stage*. The 95% CI estimates evaluated in our simulation are based on profile likelihood (“*profiled*’) corresponding to the *maxLogLike* estimator, the two-stage method (*varCI)* corresponding to the *2stage* estimator, and $CI_{Delta}$ based on Equation (6) of Section 3.3, with each of the three types of the point estimators described in Section 3.4.

The simulations covered a wide range of scenarios with some of the parameters (e.g., slope and variance parameters) chosen to reflect our motivating example as presented in Figure 1:

1. Number of subjects: N=100,200,300,400,500.
2. Number of longitudinal visits beyond baseline: J=3,4,5.
3. Baseline age: simulated from a truncated (between 40 and 90) normal distribution with a mean of 60 and a standard deviation of 12.5 (mimicking the real AD dataset). We also simulated age from a uniform distribution and found similar results (thus not presented).
4. The true change-point in baseline age: τ=45,55,61, and 70 years.
5. The slope parameters $\left(\beta_{1-},\beta_{1+}\right)$ were set to be (0, 0.15) while the other nuisance parameters in the fixed effects set at 0.
6. The random effects $\left(b_{0i-},\;b_{1i-}\right)∼\text{N}\left(0,\left(\begin{array}{cc} 0.015 & 0.001\\ 0.001 & 0.015 \end{array}\right)\right)$ for subjects with baseline age prior to the change-point, and $\left(b_{0i+},\;b_{1i+}\right)∼\text{N}\left(0,\left(\begin{array}{cc} 1 & 0.15\\ 0.15 & 0.1 \end{array}\right)\right)$ for those with baseline age post the change-point, again reflecting the greater variability post the change-point in our motivating example (Figure 1). **0** is a vector of zeros of dimension 2.

We simulated each data set according to Model (1), assuming J follow up visits per subject under a balanced design. Then, missing data were assumed at random, and simulated by allowing each observation within each subject to have a missing probability of 10%.

### Simulation Results

**Figure 3.**
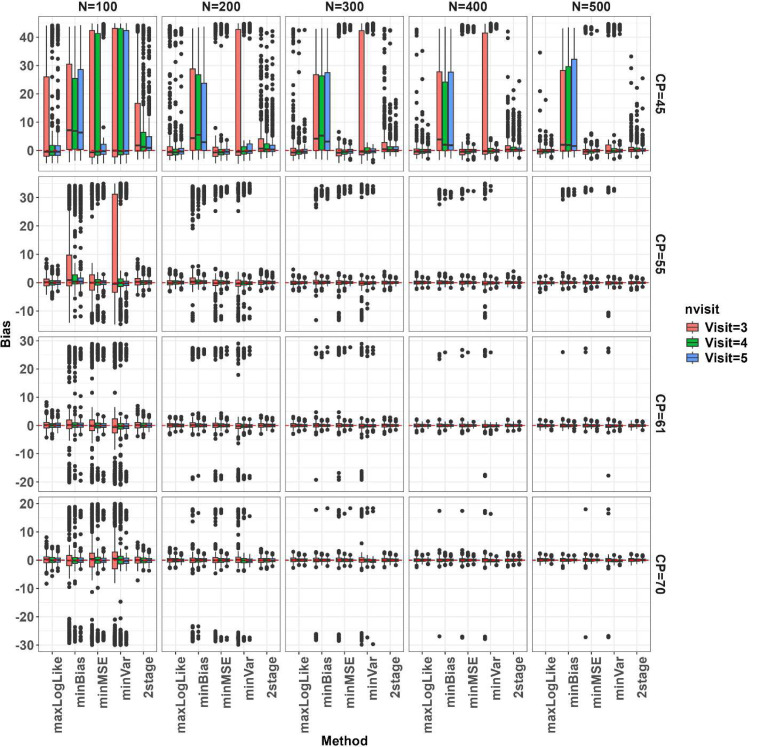
Bias of the change-point estimators across simulation scenarios in box plots. True change-point (CP=45, 55, 61, and 70 years) is indicated at the row panels, number of subjects (N from 100 to 500 by 100) at column panels, and number of follow up visits (=3, 4, 5) by red, green, and blue colors. Methods of change-point estimation are on the horizontal axis (maxLogLike, minBias, minMSE, minVar, and 2stage).

**Figure 4.**
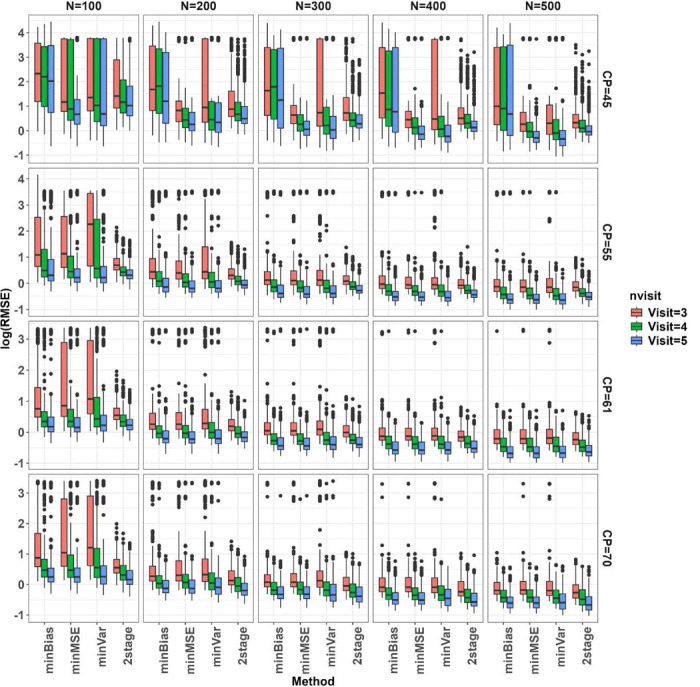
RMSE (in log scale) of the change-point estimators across simulation scenarios in box plots. True change-point (CP=45, 55, 61, and 70 years) is indicated at the row panels, number of subjects (N from 100 to 500 by 100) at column panels, and number of follow up visits (=3, 4, 5) by red, green, and blue colors. Methods of change-point estimation are on the horizontal axis (maxLogLike, minBias, minMSE, minVar, and 2stage).

**Figure 5.**
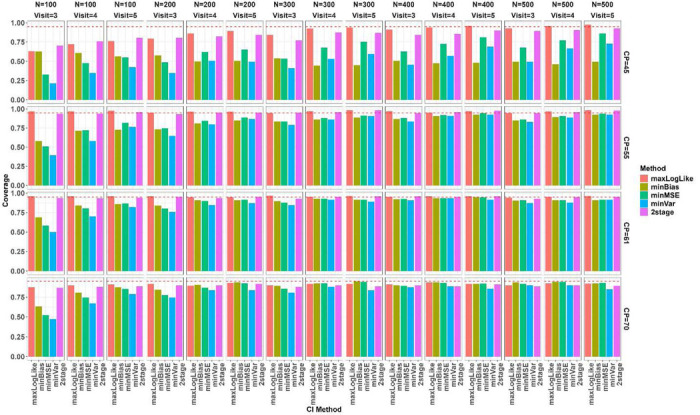
Empirical coverage of proposed 95% CIs to the true change-point under simulation scenarios. The coverage was evaluated for the CIs with the -different point estimators indicated by colors: profile likelihood CI with the *maxLogLike* estimator and Delta method-based CI with point estimators from *minBias*, *minMSE*and *minVar,* and *2stage*. True change-point (CP=45, 55, 61, and 70 years) is indicated at the row panels, and combinations of number of subjects (N from 100 to 500 by 100) and number of follow up visits (Visits=3, 4, 5) at the column panels. The horizontal red dashed line indicates the 95% coverage.

**Figure 6.**
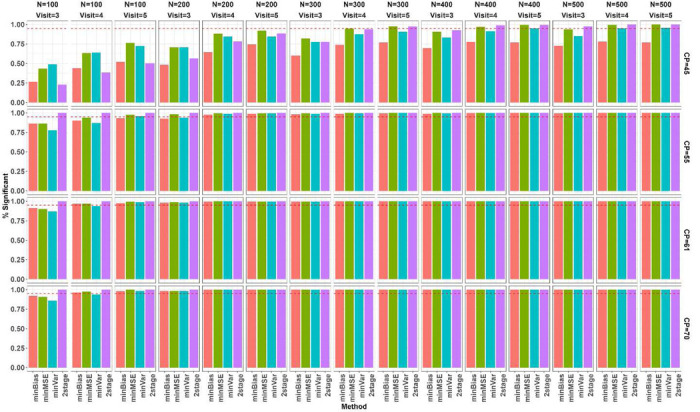
Empirical power of 400 statistical tests on existence of change-point (indicated at x-axis and by colors are the methods of estimation: minBias, minMSE, minVar, 2stage). True change-point (CP=45, 55, 61, and 70 year) is indicated at the row panels, and combinations of number of subjects (N from 100 to 500 by 100) and number of follow up visits (Visits=3, 4, 5) at the column panels. The horizontal red dashed line indicates 95% power.

We simulated 400 data sets for each combination over true change-point (CP), number of subjects $\left(N\right)$, and number of follow up visits $\left(J\right)$, and evaluated the proposed change-point estimators across the simulated datasets in terms of bias, root mean squared error (RMSE), and coverage of 95% confidence interval (CI) as presented in Figure 3, 4, and 5, respectively. The MLE from profile likelihood of Model (1) was evaluated for coverage using the 95% profile likelihood based CI. The 2-stage method was evaluated in its own variance and CI estimators as originally proposed in Muggeo^5^. Overall, performance of the methods depends heavily on where the true CP is located and the sample sizes. When the CP was located near the left boundary (at CP=45 years), *minBias* performed poorly in estimating the change-point even with large sample sizes. When N is small (=100), all the methods performed poorly, showing large biases, large RMSEs and low coverage of 95% CIs, but *maxLogLike* and *2stage* improved with more longitudinal visits. All the methods, except *minBias*, showed dramatically improved performance of detecting CP=45 years with a sample size of at least 200. When the change-point moved closer to the center of the age range, all methods seemed to perform well and comparably, with much less bias, reduced RMSE, and improved CI coverage. When the sample size increased, all methods led to unbiased estimators with reduced RMSEs, and improved CI coverage. Increasing the number of within-subject assessments did not affect much on bias, except at N=100. However, RMSE reduced gradually with the increasing number of within-subject assessments at a fixed N and the reduction was clearly seen under large Ns. The box plots in Figure 3 and 4 further indicated that change-point estimates may be extreme or sometimes even out of range for multiple of our proposed methods even with a relatively large N. However, the likelihood of extreme or outlier estimates was much reduced when N was increased. Figure 5 indicated that the 95% nominal coverage is almost achieved for change-point away from boundaries (CP=55 and 61 years) especially with large N, but not for small N with change-point close to the boundaries, though *maxLogLike* and *2stage* provided better coverage than other methods. We also assessed in Figure 6 the empirical power of detecting the existence of a change-point by calculating the percentage simulations when the statistical testing of existence of a change-point gave a p value <0.05 (with p values derived from the Davies test for the 2-stage approach and Wald test in Section 3.3 for the others). The power increased, as expected, with an increasing number of participants and number of visits. The power was low at N=100 and the boundary CP=45 years but became much improved with large N and the CP away from the boundary. For CP=45 years, *minBias* showed a relatively lower power than others even under a large N of 200 to 500.

### Identifying the baseline Age when Longitudinal Change in White Matter Hypointensities started to Accelerate

On computed tomography (CT) and T1-weighted magnetic resonance imaging (MRI), white matter lesions appear dark and have been termed as white matter hypointensities (WM-hypo), while on fluid-sensitive MRI sequences such as fluid-attenuated inversion recovery (FLAIR), they appear bright and are referred to as white matter hyperintensities (WM-hyper). WM-hyper and WM-hypo are highly correlated^2^, and both are associated with other validated biomarkers of AD and cognitive decline ^2,3^. Whereas WM-hyper has been well reported to increase with age and predict an increased risk of dementia and cognitive decline^17,18^, WM-hypo was much less studied, especially with longitudinal assessments, which is the focus of this application.

Our longitudinal data of WM-hypo are from the Washinton University Knight AD Research Center (ADRC). One of the major scientific aims of the Knight ADRC is to detect the earliest possible biological changes and dementia symptoms associated with AD. Both cognitively normal middle aged and old individuals and those diagnosed with early symptomatic stages of AD dementia were enrolled for longitudinal clinical and cognitive assessments. Every two to three years, participants also underwent MRI and other imaging (e.g., positron emission tomography) assessments. MRI brain scan acquisition and processing were previously described^17^. The WM-hyper (on fluid-attenuated inversion recovery scans) and WM-hypo were quantified using the SPM8 lesion segmentation toolbox^19^.

Because changes in many biomarkers of AD start many years prior to the onset of clinical symptoms^1^, the asymptomatic stage becomes the most crucial time window for designing clinical trials to test potential AD prevention therapies. An important inclusion criterion of these trials is the baseline age window that indicates an elevated risk of AD progression. We therefore focused on 616 Knight ADRC participants who were cognitively normal at baseline, as defined by a global Clinical Dementia Rating^®^ (CDR^®^)^20,21^ of zero, and located a change-point in baseline age that is associated with an accelerated rate of longitudinal change in WM-hypo. Among the 616 participants, 240 participants had 2 longitudinal follow up visits, 160 had 3 visits, 88 had 4 visits, 59 had 5 visits, and 69 had at least 6 longitudinal data points. The baseline age distribution of the participants was shown in Supplemental Figure 1. The longitudinal trajectories of WM-hypo across the participants as functions of age at longitudinal follow-up were shown in Figure 1. A visual examination of Figure 1 indicated a potential baseline age change-point between 60 and 75 years when the longitudinal rates of change in WM-hypo started to accelerate. We set the search grid of baseline age between 50 and 80 with an increment of 0.01 to identify a potential change-point using the proposed methods in this paper.

**Figure 7.**
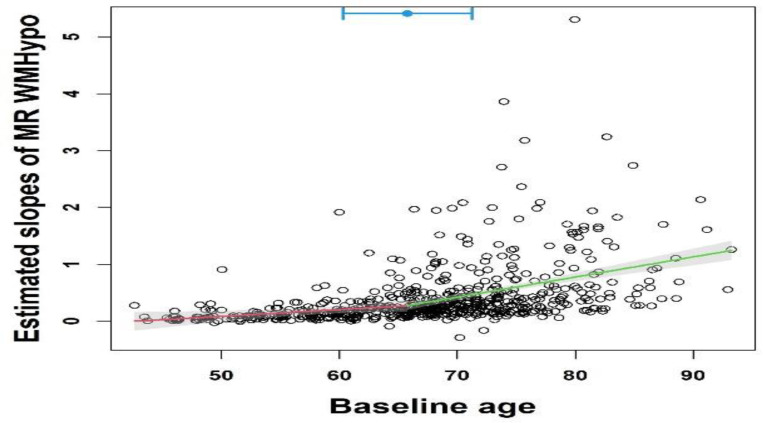
A change-point of 65.78 years identified from the two-stage model of longitudinal rates of change in WM-hypo (Davies test in Muggeo ^5^: p-value=0.004). Horizontal point and line at top indicate the estimate with the associated 95% CI.

The estimates for the change-point were presented in Table 1. The estimate from maximizing the profile likelihood (*maxLogLike*) was 79.7 years, near the maximum boundary of the search grid. Minimizing Delta method-based variance (*minVar*) yielded a much earlier change-point estimate at 59.19 years, and minimizing mean square error (*minMSE)* led to a close estimate at 60.37 years. Estimates from minimizing the squared pseudo bias (*minBias*) and the two-stage approach were similar (Table 1), at around 63.29 years and 65.78 years (Figure 7), respectively. For the proposed 95% CIs to the change-point, profile likelihood-based CI was very narrow (79.06 ~79.8 years), and the other 95% CI were similar in length. Given that the existence of a change-point is the most important assumption in Model (1), we further sought to statistically confirm a change-point, beyond a simple visualization, by comparing the slope of WM-hypo (i.e., per year longitudinal change) among participants with baseline age prior to and post the estimated change-point. The Davies test (see Section 3.5) from the two-stage method gave a p-value of 0.004, the Wald test (Section 3.3) and other methods all led to a p-value of p<0.0001 (Table 1), all supporting the existence of a change-point. In summary, whereas all other methods led to close change-point estimates, an extreme and outlier estimate was given by the profile likelihood approach. This is consistent with what we observed in our simulation study, especially with small sample sizes. Because the simulation study suggested that the likelihood of extreme and outlier estimates decreased as the sample size increased, it is possible that a much larger sample size is needed (than the current sample size in our AD study) to avoid the extreme estimate from the MLE through profile likelihood.

## Discussion

This paper addressed a statistical challenge arising from a real-world biomarker study of AD. Because age is the most important risk factor in AD, older individuals have a higher risk for AD dementia. Prevention trials on AD are conducted on individuals who are cognitively normal but at high risk of AD, and hence must determine at what minimum age, participants should be enrolled in the trials. One important consideration is to find the age when cognitively normal individuals start to show an elevated risk of AD. Because imaging biomarkers of AD are all functions of age, we conceptualized that an elevated risk of AD may be represented by a change-point of age so that older individuals (than the change-point) at baseline will exhibit an accelerated longitudinal rate of change than the younger individuals in these biomarkers. This simple conceptualization not only makes clinical sense but is also well supported by the real-world biomarker study of AD in which longitudinal changes in WM-hypo, as an important biomarker of AD, clearly demonstrated an accelerated longitudinal increase as a function of baseline age (Figure 1).

The statistical challenge is then how to best estimate the change-point. Although AD is a decades-long neurodegenerative disease, most of the biomarker studies of AD have thus far accumulated longitudinal data only over a relatively short time window with a limited number of repeated measurements (2, 3, or 4 visits), partly due to the fact that many of the AD biomarkers are only recently developed and costly, and some of the procedures may also be considered invasive by participants. The limited longitudinal follow up (both in duration and frequency of assessments) prevented us from comprehensively analyzing the potential nonlinear longitudinal trajectories over the entire disease course that could last decades and identifying a change-point in the middle of longitudinal follow up. We therefore opted to fit the random intercept and random slope models that reasonably captured mostly linear longitudinal changes within individuals (Figure 1), but leveraged on the wide window of baseline age that spanned from mid 40s to almost 100 years to assess how it predicted the longitudinal changes in the biomarker (Figure 1). Our models accounted for these important design and analytic features by conceptualizing a change-point in baseline age on the longitudinal rate of change in the biomarker. Specifically, the change-point was defined by baseline age when the longitudinal rate of change in the biomarker started to accelerate, allowing the longitudinal rate of change as a nonlinear function of baseline age. Our models are in contrast to several longitudinal change-point models that were proposed in the literature, especially for modeling longitudinal cognitive scores whose primary goal was to identify a change-point in the middle of longitudinal follow up where accelerated cognitive decline^7–11^ occurred. These types of change-point models typically require longitudinal data with a very long follow-up and many within-individual repeated assessments over time because there must be adequate longitudinal data both prior to and post the change-point. Whereas these methods are adequately applied to the analysis of extensive longitudinal cognitive data (which are currently available in many AD studies) ^7–11^, they do not directly apply to most biomarker studies in AD from which only short-term longitudinal data with limited follow up are currently available. Further, our incorporation of the baseline age change-point in the slope led to a unique analytic challenge because the likelihood function is not a smooth function of the model parameters, which then questions the validity of standard MLE-based asymptotic inferences, especially about the change-point, the main interest of our study. Given that change-point models have been well studied in the literature under a cross-sectional design with regression models^5,6^, our proposed model can be viewed as a bridge between the cross-sectional change-point models and longitudinal change-point models that require extensive longitudinal follow up in the current literature. Importantly, modeling the annual rate of change directly as a piecewise linear function of baseline age reflects the fundamental biomedical science that age is the biggest risk factor of AD^22^ and associated with AD biomarkers^3,23,24^, and facilitates the establishment of age-related inclusion/exclusion criteria for designing AD prevention trials.

We provided multiple methods to estimate the change-point and constructed the corresponding CIs. Because these methods require rather intensive computations, we provided specific algorithms to implement them. We further evaluated the performance of the proposed estimators through an extensive simulation study, assessing how the sample sizes, the frequency of longitudinal follow-ups, and the location of the change-point (i.e., close to vs. away from the boundary) may affect the performance. We found that performance was universally poor for all point estimators and CI estimates when the true change-point was near the boundary or when sample size was small, say, N=100. Yet, the proposed change-point estimators became approximately unbiased and showed relatively small RMSEs when sample size increased (at least N=200) and the true change-point was away from boundary. The 95% CIs from these methods also provided good nominal coverage with large sample sizes if the change-point was away from boundary. However, our proposed estimators for the change-point may result in extreme or out of range estimates or outliers when the sample size is too small and the change-point is too close to the boundary, but the likelihood was markedly reduced with much larger sample sizes. Given these observations, we do not recommend applying these models to estimate a change-point for data sets with less than 200 subjects, or when the change-point is located near the boundary. For the latter, we recommend exploratory analyses first to visualize a likely age window for a change-point through spaghetti plots (as in Figure 1) by implementing the two-stage model to further visualize the estimated subject-level slopes against baseline age in scatter plots. Setting a search grid for the change-point based on the likely age window from the exploratory analyses will also help locate the change-point more efficiently and increase confidence in the estimation results. Finally, one should always confirm the existence of a change-point by comparing the longitudinal slopes post and prior to the estimated change-point, which can be based on a Wald test (Section 3.4) or the Davies’ test in the 2-stage model (Section 3.5).

We re-visited the real-world AD biomarker study that motivated this work and found that there indeed existed a change-point of baseline age in the annual rate of change of WM-hypo. Almost all methods yielded similar estimates to the change-point from 59.19 years to 65.78 years, but the profile likelihood approach led to a much later estimate of 79.7 years. Specifically, the profile likelihood in Supplemental Figure 2 indicated the existence of several local maxima, suggesting the possibility of multiple change-points in baseline age that begs for further research in this direction. On the other hand, this finding seems to be consistent with the simulation results that indicated extreme estimates could occur in our proposed methods, and larger studies are needed to best estimate the change-point in WM-hypo. Nonetheless, the estimated change-point from 59.19 years to 65.78 years from most of our proposed methods is consistent with the visualization in the spaghetti plot (Figure 1) and may provide age windows that longitudinal increase in WM-hypo among cognitively normal individuals started to accelerate, hence informing future prevention trials on the possible age-based inclusion/exclusion criteria, especially if WM-hypo is the target of the prevention.

## Conclusion

We have proposed a longitudinal model that accounted for an accelerated rate of longitudinal change as a function of baseline age to address a real-world statistical challenge from a biomarker study of AD. We provided multiple approaches to estimate the change-point and derived their CIs. Simulation studies and the real-world application indicated that these estimators performed reasonably well, especially when the change-point is away from the boundary and the sample sizes are large. However, further research is needed to tackle more complex challenges in this line of research. For example, our model only allowed a single change-point, but the progression of AD spans over decades, and it is likely multiple change-points may exist in the process. Further, change-points may also depend on covariates, such as sex, *APOE* genotypes, and racial and ethnic groups which are well established risk factors of AD^3^‘^23,24^. These extensions require larger sample sizes and more complex statistical models.

## Supplementary Material

Supplement 1

## Figures and Tables

**Table 1.** Estimates of change-point for WM-hypo and the associated 95% CIs

Method	Change-point Estimate (in years)	95% CI*
maxLogLike	79.70	79.06 ~79.80
minBias	63.29	58.16~68.43
minVar	59.19	55.34~63.05
minMSE	60.37	56.14~64.60
two-stage	65.78	60.32~71.25

* The profile likelihood-based CI is reported for the *maxLogLike* method. For the 2-stage approach, the 95% CI for the change-point estimate was based on Delta method-based variance derived in Muggeo ^5^.

## Data Availability

Access to the data set used in this paper can be requested online (https://knightadrc.wustl.edu/professionals-clinicians/request-center-resources/submit-a-request/), or to the first author (chengjie@wustl.edu).
