# Supplementary material for: Estimating a change-point of baseline age in the longitudinal trajectories of biomarkers: application to an imaging study of preclinical Alzheimer disease
Source: Res Sq. 2025 Jun 12:rs.3.rs-6681661. Preprint. [Version 1] doi: 10.21203/rs.3.rs-6681661/v1 (PMC12204498; doi:10.21203/rs.3.rs-6681661/v1)
Supplement: Supplement 1 [file NIHPPRS6681661V1-supplement-1.pdf]

Supplemental Figure 1. Distribution of N=616 participants' baseline age in the AD biomarker study dataset.

Supplemental Figure 2. Log profile likelihood as a function of age from the search grid of 50 to 80 years for the change-point.

Supplementary Files

This is a list of supplementary files associated with this preprint. Click to download.

- StatisticalInferenceonLongitudinalChangePointSupplementalFigures5152025.docx
